# Chronic kidney disease of nontraditional causes in central Panama

**DOI:** 10.1186/s12882-022-02907-3

**Published:** 2022-08-05

**Authors:** Karen Courville, Norman Bustamante, Bárbara Hurtado, Maydelin Pecchio, Clarissa Rodríguez, Virginia Núñez-Samudio, Iván Landires

**Affiliations:** 1 Instituto de Ciencias Médicas, PO Box 0710-00043, Las Tablas, Los Santos Panamá; 2 Unidad de Hemodiálisis, Departamento de Nefrología, Hospital Dr. Gustavo N. Collado, Caja de Seguro Social, Chitré, Herrera Panamá; 3 Departamento de Epidemiología, Hospital Dr. Gustavo N. Collado, Caja de Seguro Social, Chitré, Herrera Panamá; 4 Unidad de Infectología, Hospital Dr. Gustavo Nelson Collado, Caja de Seguro Social, Chitré, Herrera Panamá; 5 Departamento de Infecciones Nosocomiales, Hospital Dr. Gustavo N. Collado, Caja de Seguro Social, Chitré, Herrera Panamá; 6 Sección de Epidemiología, Departamento de Salud Pública, Región de Salud de Herrera, Ministerio de Salud, Chitré, Herrera Panamá; 7 Hospital Joaquín Pablo Franco Sayas, Ministerio de Salud, Las Tablas, Los Santos Panamá

**Keywords:** Chronic kidney disease, Panama, Nontraditional, Traditional, Mesoamerican nephropathy, Clinical presentation

## Abstract

**Background:**

Over the last three decades, the mesoamerican region has seen an increase in the frequency of patients diagnosed with Chronic Kidney Disease of nontraditional causes (CKDnt) also known as Meso-American Nephropathy (MeN). A region with an increased frequency of patients with Chronic Kidney Disease (CKD) has been identified in central Panama. The present study aims to characterize the clinical presentation of patients with CKDnt in an understudied population of the central region of Panama and to compare them with patients with traditional chronic kidney disease (CKDt).

**Methods:**

A retrospective descriptive study was conducted in a nephrology reference hospital in the central provinces of Herrera and Los Santos, comparing a group of 15 patients with CKDnt to 91 patients with CKDt. Sociodemographic variables, personal history, laboratory parameters, and of renal ultrasound were compared.

**Results:**

Patients with CKDnt had a median age of 58 years (IQR: 52–61), significantly lower (*P* < 0.001) than patients with CKDt with a median age of 71 years (IQR: 64–78). Patients with CKDnt had a history of being agricultural (60%) and transportation (20%) workers, significantly higher than patients with CKDt (15%, *P* < 0.001 and 0%, *P* < 0.01 respectively). Renal atrophy and hyperuricemia are significant clinical markers of CKDnt (*P* < 0.001 and *P* < 0.05 respectively).

**Conclusion:**

To our knowledge, this is the first study in Panama to investigate the clinical presentation of patients with CKDnt and one of the few in Central America and the world that compares them with patients with CKDt. In central Panama the typical CKDnt patient is a male in his 50 s who is primarily engaged in agriculture or as a public transport driver. Renal atrophy and hyperuricemia are significant clinical markers of CKDnt. Further studies are needed to help understand the common determinants and risk factors for CKDnt development in Panama and Mesoamerica.

## Background

Chronic Kidney Disease (CKD) belongs to the group of non-communicable diseases and, according to estimates from the Global Burden of Disease study, led to a 19.6% increase in disability-adjusted life years (DALYs) and has been associated with 4% of deaths worldwide between 2005 and 2015, representing 2.2 million deaths per year [1, 2].

The cause of CKD is identified in 60% of patients. Traditional causes of CKD include diabetes mellitus, essential hypertension and obesity, followed by a number of other minor causes, such as immunological diseases, nephrolithiasis and genetic conditions [3]. Approximately 15% of adult patients with CKD may have a family history of kidney disease. In 10% of patients, the cause of CKD cannot be identified and is classified as unknown or non-traditional cause [4]. Since the early 2000s, an increase in the frequency of diagnoses of Chronic Kidney Disease of non-traditional cause (CKDnt) has been identified in patients from agricultural areas of Central America (El Salvador, Nicaragua, Guatemala, and Costa Rica), and since it was described in the Mesoamerican region, it was denominated Mesoamerican Nephropathy (MeN). CKDnt was mainly observed in young male patients with irreversible deterioration of renal function. Among the hypotheses proposed, it has been debated that chronic exposure to heavy metals, pesticides, working conditions with exposure to high temperatures and dehydration, chronic use of anti-inflammatory drugs, high alcohol and tobacco consumption, could be risk factors for the development of this disease [5, 6]. One of the reasons why it has been difficult to discover the etiology of CKDnt is that most of the available data comes from hospital records or from agricultural communities. Population-based studies are mainly limited.

The Pan American Health Organization (PAHO) defined patients with CKDnt as those with impaired renal function with a glomerular filtration rate (GFR) of less than 60 mL/min/m^2^ [7], in the absence of predisposing factors of traditional CKD (type 2 diabetes mellitus, essential hypertension, heart disease, urinary tract malformations, immunological and congenital diseases). In addition, the diagnosis of patients with CKDnt includes kidney damage defined by structural abnormalities (i.e., renal atrophy without obstructive pattern) or urinary sediment abnormalities and exposure to occupational risk factors or living in a risk area [8].

In Panama, since 2014, an increase in patients meeting the criteria for CKDnt have been reported in the central provinces of Coclé, Herrera, and Los Santos [9]. In 2017, Panamanian health authorities established the CKD Epidemiological Surveillance Information System to procure a mandatory report of all patients diagnosed with CKD at all stages and to identify associated risk factors [10]. The system aims to diagnose and establish the up-to-date prevalence of CKD in Panama to inform public policies for the promotion, prevention, and treatment of CKD. The 2017 Preventive Health Census of the Ministry of Health estimated the national prevalence of CKD at 3.24% [11].

The aim of this study is to characterize the clinical presentation of patients diagnosed with CKDnt in the provinces of Herrera and Los Santos, in the central region of Panama, and to compare them with patients with CKDt from the same geographical area.

## Methods

A retrospective and descriptive study was conducted in the Nephrology Department of the Hospital Dr. Gustavo N. Collado. This hospital is the nephrology reference center for Central Panama (provinces of Herrera and Los Santos, serving an estimated population of 214,624 inhabitants in 2020) [12]. Following approval of the study by the research bioethics committee, the investigators reviewed the records of patients who had at least two evaluations 3 months apart in the nephrology outpatient clinic between 1 January and 31 December 2018. Data on personal history, sociodemographic variables, blood samples, urine examination and renal ultrasound evaluation were obtained. This study included patients diagnosed with CKD with a calculated GFR of less than 60 ml/min/m2 using the Chronic Kidney Disease Epidemiology Collaboration (CKD EPI) formula estimation, after achieving a stable creatinine value, with an interval of at least 3 months, to establish the degree of chronicity and to be able to rule out acute renal failure [13]. Patients were classified as CKDnt if they met the diagnostic criteria proposed by PAHO [8]. Patients who had predisposing factors for traditional CKD were classified as CKDt.

Data was extracted into MS Excel (The Microsoft Corporation; Redmond, WA) and the cleaned dataset was exported to Stata v. 11.0 (StataCorp, LLC; College Station, TX) for analysis. Analyses included descriptive statistics. Due to the small sample size, Fisher's exact test was used to compare proportions and the Mann–Whitney U test was used to compare medians, setting an alpha value of 0.05 for statistical significance when comparing the frequencies of the CKDt and CKDnt groups.

## Results

**Fig. 1:**
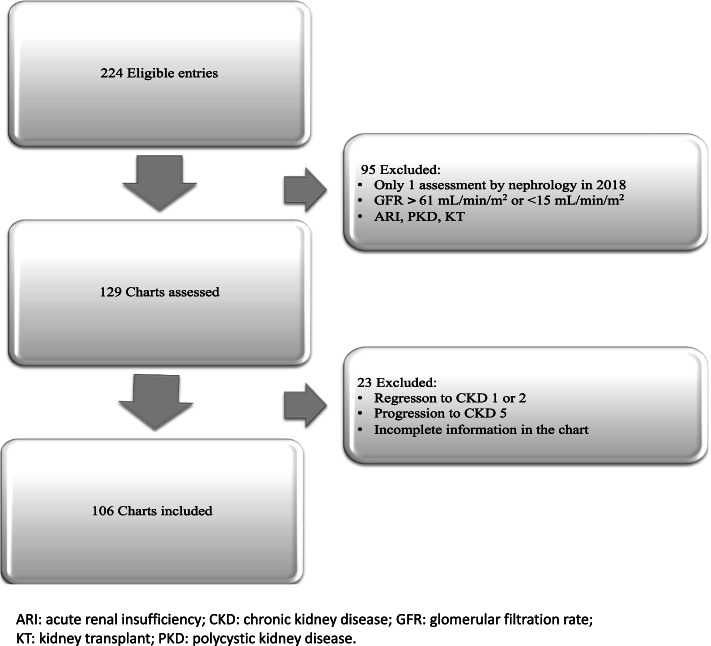
Patient chart selection flow diagram

The evaluation of the data of the medical ambulatory diagnostic system identified 224 medical records of patients diagnosed with stage 3 and 4 CKD during 2018. After reviewing the inclusion criteria, 106 patient records were included (Fig. 1).

Of the 106 patients included, 66% (70) were male and 34% (36) were female, with a median age of 69 years (IQR: 60–76). Forty-five percent of the patients were 70 years of age or older. Median weight was 73.25 kg (IQR: 62–85), median height was 1.63 m (IQR:1.55–1.69) and median Body Mass Index (BMI) was 27.37 kg/m^2^ (IQR: 24.14–32).

In the evaluation of work history, 22% of the patients were retired, followed by 20% agricultural workers, 19% household administrators, 14% unemployed, and 25% practiced other professions. Eighty percent of the patients had a history of essential hypertension, 30% of type 2 diabetes mellitus, 19% of cardiovascular disease, 15% of hyperuricemia, and 10% of cerebrovascular disease.

Fourteen percent of the patients (*n* = 15) met the definition of CKDnt, while 86% (*n* = 91) were diagnosed with CKDt. Patients with CKDnt had a median age of 58 years (IQR: 52–61) and patients with CKDt had a median age of 71 years (IQR: 64–78). Regarding gender distribution, 93% of patients with CKDnt were male versus 61% of patients with CKDt who were male (*P* < 0.05). In addition, patients with CKDnt were mostly agricultural or transportation workers, with significantly (*P* < 0.001) higher frequencies for these occupations than in patients with CDKt. Although not statistically significant (*P* = 0.09), patients with CKDnt tended to have a lower BMI than patients with CKDt.

When personal pathological history was evaluated, 20% of patients had cardiovascular disease, 11% of patients had cerebrovascular disease and none of the patients in the CDKnt group had this pathological history. Eighty-eight percent of the patients with CKDt had essential hypertension and, in comparison, only 33% of the CKDnt group had a history of hypertension that we consider non-essential because it has less than five years of evolution and has developed as a consequence of the impaired renal function.

Renal ultrasound findings were described as normal, bilateral renal atrophy, hydronephrosis or renal agenesis. Of note, ultrasonography confirmed that renal atrophy was significantly more frequent (*P* < 0.001) among patients with CKDnt than among patients with CKDt. Patients diagnosed with CKDnt showed significantly lower blood levels of glucose (*P* < 0.05) and higher blood levels of uric acid (*P* < 0.05) than patients diagnosed with CKDt.

Table 1 summarizes the relevant clinical, history, ultrasound, and laboratory data for both groups.

**Table 1:** Relevant Clinical and Paraclinical Data of patients with CKDnt and CKDt

**Characteristics**	**CKDt (*n* = 91)** Median (IQR)	**CKDnt (*n* = 15)** Median (IQR)	***P* value**
Age, years	71 (64–78)	58 (52–61)	< 0.001
Weight, kg	73.5 (61–87)	70 (63–76)	0.59
Height, m	1.62 (1.55–1.68)	1.67 (1.57–1.69)	0.13
BMI, kg/m^2^	27.38 (24.7–32.35)	26.61 (22.4–28.6)	0.09
Gender	**n (%)**	**n (%)**	0.016
Men	56 (61)	14 (93)	
Women	35 (38)	1 (7)	
Agricultural workers	14 (15)	9 (60)	< 0.001
Transportation workers	0 (0)	3 (20)	< 0.001
Personal history of:			
CKD	17 (19)	2 (13)	0.80
Obstructive uropathy	9 (10)	0 (0)	0.40
Hyperuricemia	12 (13)	4 (27)	0.37
Renal ultrasound^a^			
Normal findings	28 (31)	2 (13)	0.16
Renal atrophy	36 (50)	13 (87)	< 0.001
Hydronephrosis	7 (10)	0 (0)	0.27
Agenesia	1(1)	0 (0)	0.68
Urinalysis			
Erythrocituria > 3 RBCs/field	8 (9)	1 (7)	0.78
Proteinuria > 3 +	15 (16)	1 (7)	0.32
Blood chemistry values	Median (IQR)	Median (IQR)	
Glucose 1, mg/dL	101 (92–121)	96 (93–106)	0.24
Glucose 2, mg/dL	105 (94–129)	97.5 (91–104)	0.04
Glycated hemoglobin A1c, %	6.2 (5.3–7.9)	5.01 (4.51–5.5)	0.11
Creatinin 1, mg/dL	1.73 (1.45–2.17)	2.1 (1.51–2.52)	0.10
Creatinin 2, mg/dL	1.76 (1.43–2.27)	1.8 (1.64–2.7)	0.13
Blood urea nitrogen, mg/dL	25.55 (18.2–33)	24.6 (21.9–26)	0.84
Uric acid 1, mg/dL	6.8 (5.4–7.9)	7.8 (7.1–8.4)	0.04
Uric acid 2, mg/dL	7 (5.9–7.8)	8.1 (6.3–9.2)	0.09
Sodium, mEq/L	141 (138–142)	139 (138–141)	0.20
Potassium, mEq/L	4.5 (4.1–5)	4.6 (4.1–5)	0.77
Calcium, mg/dL	9.75 (9.2–10)	9.7 (9.4–9.7)	0.72
Triglycerides, mg/dL	151.5 (117–203.5)	154 (112–203)	0.90
Total cholesterol, mg/dL	189 (165–210)	175.5 (125–185)	0.14
HDL cholesterol, mg/dL	45 (38–56)	45 (37–62)	0.83
LDL cholesterol, md/dL	123.5 (94.5–144)	109 (75–125)	0.59

^a^Only 72 of the CKDt files had renal ultrasound on file

*BMI* Body mass index, *CKDt* Traditional chronic kidney disease, *CKDnt* Non-traditional chronic kidney disease, *IQR* Interquartile range, *RBC* Red blood cells, *HDL* High-density lipoprotein, *LDL* Low-density lipoprotein

## Discussion

This study describes the clinical presentation of patients diagnosed with CKDnt compared to CKDt in central Panama. In the group of patients with CKDnt, a male predominance was observed compared to the group with CKDt. This has also been observed in other countries, such as El Salvador (78%) [14], Nicaragua (77%) and Costa Rica (70%) [15]. The median age was lower in the CKDnt group, compared with the median age in the CKDt group. The latter is similar to what was observed in the study group from agricultural communities in El Salvador, where the median age of patients was 45 years [14]. The increased frequencies of patients with diagnoses of CKDnt have been observed mainly in some defined areas in Central America. In particular, these are areas with high agricultural employment, where young labor is hired to perform difficult tasks under the tropical sun. These factors may explain, at least in part, the younger age, lower BMI and higher prevalence in males presenting with CKDnt. In turn, these patients did not have chronic diseases typically associated with CKDt, such as type 2 diabetes mellitus and essential hypertension [16]. This study showed that a significantly higher proportion of patients with CKDnt had a history of agricultural work compared to patients in the CKDt group. The central region of Panama has the largest area of corn cultivation in the country, with more than 26,000 hectares per year. The province of Herrera is the fourth in sugarcane production nationwide [17]. The patients in this study belong to the central provinces of Herrera and Los Santos, mostly sea level land dedicated to agriculture, in a peninsular area that frequently exhibits the highest temperatures in the country of up to 35 °C, especially in the dry season between December and April [18]. It has also been described that in Panama there has been a temperature increase of up to 1 °C between 2010 and 2015 and that this has been significant in the central provinces of Herrera and Los Santos. Predictive models have been proposed for a temperature increase of up to 15% in the coming decades in the central region of Panama [18]. This could contribute to the increase in CKDnt cases compared to CKDt causes in recent years, as a consequence of continuous dehydration due to exposure to high temperatures during strenuous work days, as has been studied in other groups of sugarcane growers in Central America [19, 20]. Second, in this study, other employment occupations, as public transport drivers were affected with CKDnt, which could also be associated with exposure to high temperatures while driving and not drinking enough water due to the inaccessibility of restrooms due to their type of work.

Previous studies have observed that arterial hypertension is rare in patients with CKDnt and has a high prevalence in CKDt patients [21]. Hypertensive nephrosclerosis is a chronic disease that gradually and progressively produces chronic renal involvement [22], and is considered in patients with long-standing hypertension of more than 5 years of evolution. The presence of mild hypertension of less than 5 years of evolution, associated with Chronic Kidney Disease, in the absence of traditional risk factors, but with association of occupational exposure, should make us suspect CKDnt. The finding of mild hypertension in some patients with CKDnt in this study should be considered a consequence of impaired renal function and, therefore, should not be a reason to rule out these patients [23], and above all should help the physician to monitor the need for medical treatment.

In this study, uric acid values showed a significant elevation above the normal value in the CKDnt group of patients compared to CKDt. In dehydration states, fructose is endogenously produced from glucose, leading to uric acid generation, inflammation and fibrosis in the kidney [24]. In addition, asymptomatic hyperuricemia has been associated with the development of mild hypertension, which, when treated and normalized, improves blood pressure control [25]. The present study supports the concept that hyperuricemia is a biomarker of CKDnt. Further studies are needed to determine its role in the diagnosis, progression, and prognosis of CKDnt.

In the evaluation of imaging studies, by renal ultrasound, most of the patients diagnosed with CKDnt presented a decrease in the cortico-medullary ratio with increased echogenicity and renal atrophy. This translates into renal morphological damage observed in early stages of asymptomatic patients. Renal ultrasound studies performed in El Salvador have found increased echogenicity up to 95% and decreased cortico-medullary ratio in up to 82% of patients with CKDnt [14]. The present study supports the concept that renal atrophy in early chronic disease stages is a clinical marker of CKDnt. Further studies are needed to determine its role in the diagnosis, progression, and prognosis of CKDnt.

This entity has different names depending on the region, either as CKDnt, CKD of unknown cause (CKDu) or Mesoamerican Nephropathy (MeN), which further complicates its epidemiological traceability [26]. Therefore, it is essential not only to diagnose it, but also to code it consistently as the same diagnosis.

A limitation of this study is the small sample size in the case of patients with CKDnt. Therefore, interpretation of its implications, including risk factor considerations, should be prudent. Another limitation of this study is that it is not population-based and therefore suffers from referral bias. The low number of cases and the probable lack of representativeness of hospital cases/controls is an important limitation of this study that should be highlighted so that any conclusions that may be drawn should be taken with caution. Another limitation is that not all patients had renal ultrasound to compare the results. Despite its limitations, this study makes important contributions to the understanding of the clinical presentation of CKDnt in patients from an endemic region.

## Conclusions

To our knowledge, this is the first published study in Panamanian patients with CKDnt, demonstrating that, as in Mesoamerican countries, the risk factors for this disease are also present in central Panama. This study is also novel because it makes a comparison of the clinical presentation between patients with traditional and non-traditional Chronic Kidney Diseases, showing in Panamanian patients the clinical features of both conditions that are useful for differential diagnosis.

This work confirms the results of other previous studies that show that CKDnt is more common in younger male patients than in those suffering from CKDt. This study also showed that in Central Panama, CKDnt is most prevalent in agriculture workers, followed by public transport drivers, thus concluding that this is an occupational disease with common risk factors to these professions that require further investigation. Renal atrophy and hyperuricemia are important clinical markers of CKDnt.

Further studies are needed to help understand the common determinants and risk factors for CKDnt development in Panama and Mesoamerica.

## Data Availability

All data generated or analysed during this study are included in this published article.

## Abbreviations

CKD: Chronic kidney disease
CKDnt: Nontraditional chronic kidney disease
CKDt: Traditional chronic kidney disease
DALY**s**: Disability-adjusted life years;
GFR: Glomerular filtration rate
BMI: Body mass index
